# M. Bretonneau on the Identity of Angina Maligna and Croup, with Numerous Cases, Dissections, and a Peculiar Mode of Treatment

**Published:** 1826-10-01

**Authors:** 


### VIII.

*Des Inflammations Speciales du Tissu Muqueux, et en Particulier de la Diphthérite, ou Inflammation pelliculaire, connue sous le Nom de* CROUP, *d'Angine Maligne, d'Angine Gangreneuse, &c.* Par P. BRETONNEAU, Medecin en Chef de l'Hôpital de Tours. 8vo. pp. 540, avec Planches. Paris, Juin, 1826.

THE author of this work is a distinguished physician, at Tours, a large city in France, and has excited considerable interest among our Gallic brethren, by his minute and accurate researches into the phenomena of affections of the mucous membrane lining the air-tubes and digestive canal. In the 9th Number of this Series, page 199, *et seq.* we gave a short sketch of Dr. Bretonneau's Observations on a Pustular Eruption in the Mucous Membrane of the Intestines, denominated by him DOTHINENTERITIS, and it is now our duty to lay before our readers some account of the large volume which he has just published on Scorbutic Gangrene of the Gums, Angina Maligna, and Croup—diseases which he affirms to be not merely similar but identical.

It will be remembered that in the fourth volume of the present Series, in the Numbers for January and April, 1826, pp. 261, and 590, we introduced some observations of Mr. Mackenzie of Glasgow, and Mr. Pretty of London, shewing that the inflammation and exudation of croup frequently commenced on the tonsils and fauces, thence spreading down into the œsophagus, larynx, and trachea. Mr. Pretty has related cases of fatal croup, "after, or rather in conjunction with, simple scarlet fever and malignant sore throat," which, "seemed to be owing to an extension of inflammation from the fauces to the larynx and trachea."

In the Number for October, 1825, of the Medical and Physical Journal, Dr. Gregory of London, details three cases of fatal croup, where the disease evidently began as cynanche, the inflammation spreading down into the larynx and trachea, and destroying life. Two of the cases were examined after death. In one, the croupal exudation was found lining the larynx and trachea as far as the first division of the Bronchia. In the other case, the child was getting better under the mercurial and antiphlogistic plan, but relapsed and died, apparently from exhaustion. In this instance the trachea was blocked up with a purulent secretion, and the mucous membrane was inflamed, but no adventitious membrane had been formed. The disease was therefore tracheitis rather than real croup. Dr. Gregory has brought these cases forward with the view of shewing the contagious character of croup; and as these three instances occurred in the same family, there is strong reason to believe that the disease was communicated from one to the other. But these cases prove another point of great consequence—the extension of inflammation from the fauces to the air-passages—or in other words, the *occasional* identity, at least, of cynanche maligna and croup. The bearing of these notices will be immediately recognized.

A singular confirmation, and indeed anticipation of these observations will be seen in the work of Dr. Bretonneau. In Tours and the neighbouring country, an epidemic has raged, with more or less violence, since the year 1818, desolating whole families by its ravages. The disease was first observed among the soldiers of the Legion of Lavendee, who had lately returned from the Isle of Bourbon. In these it assumed the form of a scorbutic affection of the gums, with small ulcerations spreading over the mucous membrane of the mouth. At first it was thought to be purely *scorbutic*, and vegetables, vegetable acids, and other antiscorbutic remedies were given in abundance, but without the least effect. The topical application of muriatic acid removed the complaint, as if by magic.

In the same year, the ANGINA MALIGNA broke out at Tours,—a disease which very few, even of the oldest practitioners, had previously seen in that part of France. A case or two will convey a description of this affection.

*Case.* A child five years of age, in prime health, was seized with coryza, and pain in the ear, accompanied by some discharge from the auditory canal. Two days afterwards, he complained of sore throat, and some difficulty of swallowing; but supped the same evening with his usual appetite. On the third day the throat was more embarrassed, and on examination, in the forenoon, large grey patches were seen covering the tonsils, surrounded by red and tumid bases. Leeches were applied to the sides of the neck, and an emetic solution was given repeatedly. The cough now became troublesome, the voice hoarse, the breath very fetid, with eschars in the throat of a black colour. The pulse became small and quick, with dyspnœa, livid pallor of the countenance, &c. and the child died in the night.

*Dissection.* The velum pendulum palati was of a dark grey colour—and decomposition had already begun to take place in the tonsils. The eschars extended from the posterior nares to the commencement of the œsophagus, penetrating down into the glottis, where they were of a pale tint. The mucous membrane of the trachea (which, however, was only hastily examined) did not appear inflamed. There was some accumulation of mucus about the bifurcation of the bronchia.

The object of the two following cases will be obvious enough.

*Case* 2. A child, eight years of age, was brought to the Orphan Hospital, with pale and leaden complexion, depressed expression of countenance, loss of voice, quick weak pulse, fetid breath. The child had complained of sore throat for some days, together with a little difficulty of breathing. On inspection, all the soft parts about the back of the mouth were of a grey colour, and apparently sphacelating—one of the tonsils was almost detached, being only suspended by some cellular membrane. Although no hope of recovery was entertained, a sponge impregnated with hydrochloric acid, was pushed into the fauces. Next day the symptoms were surprisingly mitigated, and membraniform sloughs were detached. The same topical application was reiterated, and the child rapidly recovered. From the appearances which the fauces presented before the acid application, it was presumed that disorganization had gone on to a considerable depth in the soft parts; yet to the astonishment of the medical attendants, when the sloughs were thrown off, the mucous membrane of the parts underneath was found to be in a state of integrity.

It is hardly necessary to state that the method of treatment above described was now generally adopted, and with the best success. The following case will shew the danger of discontinuing the topical application too soon.

*Case* 3. A child, seven years of age, was brought into the general Hospital of Tours, (ward No. 4,) with sore throat, slight fever, the right tonsil a little swelled, with a white spot on it, which disappeared after the second application of the muriatic acid. Four days passed, without any thing particular occurring, the child denying that its throat was sore, lest the acid application should be renewed. But at length the difficulty of deglutition could not be concealed; and, on examination, it was found that the whole of the fauces were of a grey and marbled colour—the cough being frequent, and the expectoration copious. The topical application was renewed. Next day the voice was much affected; and the day after, it was entirely extinct. The cough was stridulous, with dyspnœa, sibilant inspiration, fetid breath, pale livid countenance. In the night the dyspnœa increased rapidly, and death took place on the third day from the relapse.

*Dissection.* The parietes of the pharynx were covered with eschars, and in the trachea was found a complete membranaceous tube, white, elastic, consistent, and feebly adhering to, or rather spread over the subjacent mucous membrane, from the rima glottidis to the minutest divisions of the bronchia. When this false membrane was removed, which required no force, the tissues underneath, both in the fauces and air-tubes, were perfectly free from any gangrenous appearance, exhibiting only some red patches, without any approach to erosion or thickening.

Here then the question presented itself, was this a case of malignant sore throat or croup? Was it a combination of the two? or was it an extension of the faucial inflammation and exudation into the larynx and trachea? Innumerable cases afterwards occurred, during the succeeding years, to prove the truth of this last conjecture.

Such were the first steps by which our author was led to an investigation that promises to be of some importance, both in a pathological and therapeutical point of view.

"Twenty-two dissections, says he, (1st Memoir) were made in succession, for the sole purpose of ascertaining if the concretions, which, in the pharynx simulated eschars, were really an inorganic substance of the same nature as the croupal exudations—and especially to ascertain whether the subjacent tissues preserved, at the same time, their integrity. Finally, it became a still more important object to determine whether, in cases proving suddenly fatal, where the breath presented no fetor, where the croupal cough and alteration of the voice had not been preceded by any difficulty of deglutition—in short, where the disease presented the most faithful picture of membranous angina (de l'angine membraneuse) whether the development of concretions had always commenced on the tonsils—on a part, in fine, where, by the aid of local treatment, one could arrest the extension of the disease." 32.

"In comparing," says M. Bretonneau, "the morbid appearances found on dissection in fifty five subjects, of all ages, who, in the course of two years, fell victims to the epidemic angina, there appeared but one instance wherein the false membrane was confined to the trachea, without any similar exudation on the tonsils or other part of the pharynx. In no case, even of the most malignant nature, was there any thing like *gangrene* of the parts. Ecchymoses of small extent, and an occasional slight erosion of those surfaces where the disease had longest continued, were the gravest alterations of structure which were ever seen. In about six or seven cases, that is, in one case in nine, the membraniform concretion extended to the ultimate ramifications of the bronchia. In about a third of the whole number it reached the great divisions of the bronchia—in all the rest it terminated at various depths in the trachea—the mechanical obstruction of the respiration appearing constantly to be the immediate cause of death in all cases." 33.

This exudation very generally pervaded the pituitary membrane of the nares, but rarely reached the external openings of the nostrils. In two young infants a membranous tube lined the œsophagus, down to the cardiac orifice of the stomach.

The above facts are contained in the first memoir of our author, read at the Royal Academy of Medicine in the year 1821. Three other memoirs follow in succession, carrying forward the investigation up to February, 1826. The substance of these we shall endeavour to grasp and compress in as small a space as possible, in the course of this article.

The following are the anatomical characters of the disease under consideration.

"It is sometimes difficult to detect the organic change, of which the concrete exudation is, in fact, the product. Most frequently it is confined to irregularly-shaped patches of dotted redness, without swelling. If examined with the microscope, the most florid of these patches will be found to be caused by a very fine vascular injection, the red puncta being minute ecchymoses, and the white interstitial points being the prominent orifices of the mucous follicles. A long narrow red streak is often seen extending to the pharynx, or descending into the trachea, either alone or accompanied by others of a similar appearance. A stripe of concrete matter forms on the middle of each of these striæ, and in the substance of these incipient concretions small semi-transparent bullæ are often seen. The edges of the pellicle are gradually confounded with the surrounding mucus which, without being altered in appearance, is changed in its quality, being no longer viscid, but coagulated in the vicinity of the concretion. These bands or stripes presently enlarge, become more dense, more homogeneous, and finally coalescing, form a complete tube, united to the subjacent mucous membrane by little elongations which dip into the orifices of the muciparous follicles. If the concretion is detached, the redness increases in the denuded points—the false membrane is reproduced, and, in proportion as the superimposed laminæ add to its thickness, it becomes more and more adherent to the organic surface beneath." 42.

After detailing some cases exemplifying the foregoing general description, M. Bretonneau remarks that "this redness of the mucous membrane without thickening of its tissue, so superficial and yet accompanied by a concrete exudation so abundant, appears to him so remarkable as to deserve the name and character of a specific inflammation, *sui generis.*" It is, he affirms, as different from common catarrhal phlogosis as malignant pustule is from herpes zoster—as different from scarlatina anginosa as scarlatina itself is from small-pox. Under this impression he begs permission to designate this phlegmasia by the term DIPHTHERITE, derived from ΔΙΦΘΕΡΑ, pellis, exuvium, vestiscoriacea.

The description, he observes, which he has given of the various aspects which this coriaceous exudation successively assumes, is often useful in a practical point of view. The long and narrow streaks which we see floating in the expectoration, when the mercurial influence is established in the system, indicates that there is not yet formed a membranaceous tube in the trachea, and that the morbid process has but recently extended to that part. Hence there is reason to hope that the morbid process itself will be modified before the tube is completely formed, and has acquired that degree of adhesion to the membrane beneath which bids defiance to all remedial measures.

At the commencement of the epidemic the death of children was generally attributed to croup, in consequence of its suddenness, and of its being preceded by the usual symptoms of that malady;—whereas, in adults, the fetor of the breath, and the livid appearance of the fauces, gave currency to the idea that cynanche maligna was the disease under which they laboured. The difference of development in the aeriferous tubes, at the different periods of life, sufficiently accounts, in M. Bretonneau's opinion, for this apparent anomaly. The fetor of breath and gangrenous aspect of the fauces, he avers, were owing to the putrid state of the concretions, and not to any gangrene of the organized structures. The exudation of blood, a common occurrence in this species of inflammation, completed the error, by giving a livid and variegated tint to the concretions which were already in a state of decomposition.

In respect to the identity of angina maligna and croup, it may be remarked that there is not much reason to wonder why physicians of former times, when dissection was so seldom performed, should have given the name of gangrenous sore throat to a disease which wears so much the appearance of gangrene. Dr. Bretonneau himself was not free from the same error—and had he not had so many opportunities of examination after death, he would still have believed that his patients died of gangrenous cynanche. But if we consider that out of more than two hundred cases of the disease attended by our author—and upwards of sixty dissections, he has only *twice* found the false membrane confined solely to the air-passages, while, in all the other instances, the disease *invariably* commenced in the pharynx, and presented, in the beginning, the symptoms of angina gangrenosa—if we consider that, in the whole of those who were examined, *post-mortem*, the false membrane which constitutes croup was found in the larynx—and finally, if we consider that the same facts, under the same forms, and attended by the same circumstances, were constantly observed by Dr. Guersent, and also by Dr. Velpeau, we shall be strongly urged to grant that angina maligna and croup are the same disease affecting different portions of the same mucous membrane.

*Causes.* It is very difficult to account for the first causes of an epidemic of any kind, even of measles, scarlatina, or small-pox. But, in whatever way the present epidemic had originated, there were proofs irrefragable that it was afterwards occasionally propagated by contagion. For these proofs we must refer the sceptical to the work itself. They appear to us quite unquestionable.

*Diagnosis.* Dr. Bretonneau takes considerable pains to point out the diagnostic symptoms by which the diphtheritic angina may be distinguished from various other phlogoses with which it is often confounded.

1. *Angina Diphtheritica.* Redness and tumefaction of one of the tonsils—seldom of the whole of both—erratic fever, not much developed—then some white spots on the surface of the affected tonsil, produced by the pellicular exudation, and easily detached—enlargement of the lymphatic glands on the sides of the neck—deglutition not very difficult, afterwards still less so—a redness surrounding the concretion, and spreading, sometimes very rapidly, to the velum palati, uvula, pharynx, and the other tonsil. Most frequently, after this sudden expansion, the progress of the diphtheritic inflammation becomes suspended for a time. The intumescence of the lymphatic glands either subsides or remains stationary—the fever is scarcely perceptible; but, after a truce of some days, or only of some hours, cough begins, and is either dry, or accompanied by some frothy expectoration:—Presently it becomes of a barking kind, indicating the propagation of the inflammation to the air-passages.

We shall not stop long to compare the above with common cynanche tonsillaris—syphilitic sore throat—scarlatina anginosa—spasmodic croup (to which it bears, for a short time, considerable resemblance)—or tracheitis, the most formidable of all the affections which simulate the diphtheritic inflammation.

2. In scarlatina anginosa, the fever accompanies or precedes the angina—the deep red colour of the velum palati and tonsils, and the swelling of these glands, precede the appearance of white specks which, by their coalescence, form the layer on the velum palati. The deglutition is at once painful and difficult—a buffy incrustation covers more or less of the tonsils. At a more advanced period, the tongue, denuded of the layer with which it was coated, assumes a deep violet tint, whether dry or moist. In the worst cases of scarlatina anginosa, the buffy crust which adheres to the tonsils becomes decomposed, changed in colour, partially detached, and then exhales a fetid odour, simulating a gangrenous affection. But even in this case, the pharyngeal inflammation is not the most dangerous part of the disease. As soon as the cutaneous efflorescence begins to turn pale, the inflammation of the throat rapidly subsides, and generally by the eighth or tenth day there is no trace of it left.

3. *Angina Stridulosa, or False Croup.* This disease is every day confounded with genuine croup, and accounts for the wonderful cures and numerous remedies that are so often puffed off in the periodical journals. This complaint presents itself often under a very formidable aspect, assuming all the features of croup in its highest degree of intensity. The following case, introduced by Dr. Bretonneau, will exemplify the disease and shew the difference between it and diphtheritis.

*Case.* At the time when angina maligna was making such havoc at Tours, a child, aged four years, which Dr. Bretonneau had seen in perfect health in the morning, was seized in the evening with the same cough, the same dyspnœa, and the same extinction of voice that had so generally preceded death in the fatal cases that were occurring at this period. *There was no swelling, however, of the lymphatic glands at the angle of the jaws, the tonsils and velum palati were free from redness or tumefaction, and no pain was complained of in the region of the larynx.* The disease therefore could not be diphtheritis. They were preparing to apply leeches, and to exhibit an emetic, when the little child fell fast asleep, and the pulse was not at all accelerated. The inspirations were stridulous, but the respiration was not quicker than natural. The cough, which was short and dry, only momentarily interrupted the child's sleep. Presently the cough became looser, and the application of the leeches was deferred. A gentle moisture appeared on the skin—the cough became more and more catarrhal; and, next morning, the complaint differed in no respect from a common cold. In another day the child was playing about, and required no farther medical aid.

Dr. Bretonneau is of opinion that the angina stridula, or false croup, is not produced or aggravated by any spasmodic affection of the glottis, but is solely caused by a catarrhal inflammation, a simple œdematous tumefaction (une simple tumefaction œdemateuse) of the mucous folds in the ventricles of the larynx.

4. *Tracheitis.* This, as was hinted above, is the most formidable imitation of angina diphtheritica, and that which requires the most prompt antiphlogistic measures. The diagnosis will not be difficult to any man of clinical experience. A case will best illustrate the difference between this disease and diphtheritis.

*Case.* N. D. aged six years, of strong and plethoric constitution, had been exposed to cold and, on the first day of the complaint, exhibited the symptoms of coryza. *2nd day.* The cough was dry, frequent, and short, with vox rauca. *The tonsils and pharynx, examined with care, presented no swelling nor spots.* The little patient complained of great pain about the larynx, which was increased by pressure: no swelling of the submaxillary glands—pulse 110. The cough became more and more husky, and soon presented a fearful resemblance to that of croup. *3d day.* Dyspnœa, quick breathing, sibilant inspirations. Twelve powerful leeches were applied to the sides of the larynx, and the bites bled most abundantly. The difficulty of breathing was relieved, but the cough remained dry and croupy as before. An ipecacuan mixture was given every hour till vomiting was produced. The cough now became looser, and assumed the character of that which accompanies tracheal or bronchial catarrh. *4th day.* There was muco-purulent expectoration, and mitigation of the fever. *5th day.* The same state. *6th day.* Convalescent.—p. 275.

### *Historical Testimonies.*

Dr. Bretonneau traces descriptions of the croupal or diphtheritic inflammation from the time of Hippocrates downwards. It is in Areteus, however, that we find unequivocal delineations of this dangerous malady.

"Ulcera, in tonsillis fiunt, aliqua mitia, aliqua pestifera, necantia. Pestifera autem sunt lata, cava, *quodum concreto humore albo, livido,*

*aut nigro sordentia.* Quod si concreta illa sordes altius descenderit; affectus ille eschara est, atque ita Græce vocatur, Latinè crusta. Crustam verò circumvenient rubor excellens et inflammatio, &c. *At si in pectus per arteriam id malum invadat, illo eodem die strangulat.* Pueri usque ad pubertatem maxime hoc morbo tentantur." This is a pretty accurate description of angina maligna spreading to the trachea and suffocating the patient. The croupal anxiety and suffocation are inimitably painted in the following passage.

"Tussis spirandique difficultas enascitur, et modus verò mortis quàm miserrimus accedit. Pallida his seu livida facies, tristantur cùm tonsillæ comprimuntur. * * * Cumque decumbunt, surgunt ut sedeant, decubitum non ferentes. Quod si sedent, quiete carentes iterum decumbere coguntur. Plerumque recti stantes obambulant, nam quiescere nequeunt. Inspiratio magna est—expiratio verò parva. Raucitas adest vocisque defectio. Hæc signa in pejus ruunt cum subito in terram collapsis anima deficit."

Dr. Bretonneau asks, can any one fail to recognize in this animated description, the very disease which forms the subject of his work? We cannot afford space to follow our author in his researches downwards to our own times; but must refer the reader who wishes for historical information on this point to the work itself, from page 57 to page 83.

Of the *occasional* transmission of the disease by means of contagion, our author has no doubt—and this was the creed of all the writers on this disease in the 17th century. The number of instances, however, in which the disease could be traced to contagion bore a very small proportion to those where a general epidemic influence was the ostensible cause of the malady.

*Treatment.*

Dr. Bretonneau believes that real diphtheritic inflammation or croup is rarely susceptible of a natural cure, in consequence of the constant tendency of the coriaceous inflammation to spread along the mucous membrane, without leaving the parts originally occupied. The rapidity with which the disease expands itself over parts so essential to life, has given rise to a multiplicity of remedies administered in a tumultuous manner, and too often without success. Our author avers that the fatality of croup is not owing to its violence, its destructive activity, nor any fluxionary movement towards the aeriferous tubes, but to the accumulation in those tubes of an inert secretion, the product of a superficial inflammation, which accumulation ultimately destroys the function of respiration, and occasions death. It need hardly be stated that this opinion applies solely to the disease under consideration, and not to a number of minor or different affections which simulate real diphtheritic croup. It will appear as omewhat staggering proposition of the author, when we find him declare that in this disease, neither bleeding, nor leeching, nor blistering, nor vomiting, nor purging will do any good—and that topical treatment is almost the only thing to be depended on—especially at the commencement of the disease.

"I am forced," says Dr. Bretonneau, "to declare, contrary to the received opinion, that bleeding in croup has done harm, and accelerated rather than checked the spread of the coriaceous inflammation. I did not abandon this measure till after reiterated proofs of its injurious effects." The same is said of local bleeding and blistering. The topical treatment alone (with the exception of calomel internally, of which we shall presently speak) was the only thing that appeared to do any good. Various external applications are spoken of by our author, with very little approbation, till he comes to the hydrochloric acid, fumigations, and calomel.

*Hydrochloric Acid.* This acid, when applied, in a concentrated state, to a sound mucous membrane, causes a coriaceous or pellicular inflammation. A slight superficial touch blanches the epithelium, which is detached and renewed without erosion. But if the action of the acid is prolonged, or its application renewed at short intervals, it produces an ulceration covered by a whitish concretion, which requires a longer or shorter time to cicatrize. It is necessary to be aware of these facts when we have recourse to this acid with the view of modifying the diphtheritic inflammation, in order that we may not confound the phenomena of the remedial process with those of the disease. *The best plan is to let the first applications be energetic, and not too frequently repeated.* Dr. Bretonneau has tried various modes of applying the acid to the pharynx and tonsils, and the following is that which he prefers. A piece of fine sponge is to be securely fixed on the end of a flexible whalebone probang, which is to be bent to a convenient form by heating and softening it in warm water. The sponge is to be dipped in the *concentrated acid*, and then gently pressed, so as to be left just moistened with the fluid. This precaution is necessary, lest, in the convulsive movements of the palate, some acid should be diffused beyond the parts designed to be cauterized. By these means it is easy to apply the acid, and to graduate its strength by various proportions of honey. When thus diluted it spreads beyond the part with which the sponge first comes in contact, and is only to be used in this manner, when the diphtheritic inflammation has gone beyond the reach of the eye, in its progress downwards.

The first effect of this topical treatment is to give the incipient inflammation a graver aspect. The concretions appear to be thickened and more extended. In 24 hours the action of the acid will have reached its utmost boundary. If the concretions do not now appear to have extended—or if they are becoming detached, we may prognosticate that the specific inflammation is already modified—and the topical applications need only be made at lengthened intervals, and of a weaker power. For the cases illustrative of this mode of treatment, we must refer to the work itself. It will be sufficiently obvious that this practice can only apply, with any prospect of advantage, to the diphtheritic inflammation, in the mouth and fauces. When the exudation has spread into the larynx and trachea, it is, of course, beyond the reach of sponge and acid. Some other mode of treating the disease must then be pursued.

*Fumigations*. Several times, when the diphtheritis had gained the mucous membrane of the larynx;—when it was no longer possible to apply to it the concentrated or diluted acid—and before Dr. Bretonneau had experienced the beneficial effects of calomel administered internally, he tried fumigations of the hydrochloric acid. Every one knows how diffusibly irritating this acid is when in a state of vapour, and how readily it excites inflammation in the mucous membrane of the lungs. This effect is precisely that which Dr. Bretonneau wished to produce, in order to modify the specific inflammation by the substitution of one less dangerous, and more easily removed.

Five times this experiment was tried, and with success. In the sixth case it proved fatal. This procedure is, in fact, dangerous and difficult to manage. It was renounced by Dr. Bretonneau, as soon as he had found in calomel a resource equally efficacious and much less hazardous. "There are circumstances (very rare it is true) where the calomel itself may prove a poison; and in such cases it would perhaps be better to try the acid fumigations than to abandon the patient to inevitable death."

*Mercurial Treatment*. After the legion of La Vendée had departed from Tours, their barracks were occupied by the 44th Regiment, and in a few days afterwards three of the soldiers became affected with the malignant angina. In two of these, the disease was arrested early by the topical treatment already described. The third was not so fortunate, and was sent to the general hospital, where he came under our author's care. The inflammatory concretion covered both tonsils, and was found to spread out of sight beyond the pharynx. This man was 23 years of age, stout, and previously in rude health. His breath was insupportably fetid—the sides of the neck considerably tumefied—the face flushed—pulse strong and quick—frequent cough, with mucous, transparent, and frothy expectoration. Experience had, in numerous instances, proved the inefficacy of blood-letting in the disease. A mixture of equal parts of honey and concentrated acid, was applied by means of the sponge to the tonsils and adjacent parts. The tone of voice did not, as yet, indicate the formation of false membrane in the trachea; but the abundance and limpidity of the expectorated mucus left no doubt that the irritation and inflammation which precede the coriaceous exudation, had already reached the air-passages. It was therefore evident that it imported but little to modify the disease in the pharynx, while it was making progress in the trachea and bronchia. All other general modes of treatment had hitherto failed in this disease, and therefore the mercurial plan was adopted as offering the only chance of success. Three grains of English calomel were administered every hour at first. The tongue, which was covered with a whitish crust, began, in the evening, to moisten, and clean at the point—the swelling of the tonsils was rather diminished—and the fetor of the breath was gone. But the cough was become rough and croupal. Mercurial frictions on the neck, chest, and arms were directed to be employed every three hours through the night. In the morning, no mercurial affection of the mouth had yet taken place. The tongue was still further cleaned than the day before. Membraniform concretions floated in the expectoration, which was copious and semi-transparent. These shreds must have come from the trachea, as the crusts were still undetached from the pharynx. They were in the shape of narrow bands, serrated at the edges, and some of them three inches in length, by two or three lines in breadth. They had not yet acquired the consistence of those false membranes that are formed into complete tubes; but, on accurate examination with glasses, they were found to possess all the other characters of the croupy exudations. The mercurial frictions were continued, but with longer intervals, and the calomel repeated as on the preceding day. In the evening the cough was less croupy—the expectoration less copious, and more opake, or like pus; but still mixed with a considerable quantity of the croupy concretion. The stools were of a green colour. *3d day of mercurial treatment.* (6th day of the disease.) Eight frictions had now been employed, and sixty grains of calomel taken internally, but without any indication of mercurial sore mouth. The concretions were almost entirely detached from the pharynx, leaving the subjacent mucous membrane bare and in a healthy condition. The mercurial frictions were discontinued, and the calomel given every two hours. *4th day of treatment.* The pharynx is entirely clean—the expectoration thick and muco-purulent, still containing shreds of the croupy concretion. Three or four evacuations in the 24 hours. The mercury discontinued. One ounce of the strong mercurial ointment, and two drachms and a half of the calomel had been used in the course of about 72 hours. From this time the cough diminished rapidly, and convalescence advanced.

Many cases are given illustrative of the beneficial effects of the mercurial treatment, for which we must refer to the work.

Dr. Bretonneau has also a long chapter on the injurious effects of mercury in certain constitutions, and especially where patients have been exposed to wet and cold during or immediately subsequent to the mercurial influence on the system. These subjects need not detain us here, as we are familiar with such effects in this country.

Our author directs the calomel to be given in doses of one or two grains every hour, mixed with sugar, and placed on the tongue. In this manner the mercury acts topically as well as constitutionally, and on the digestive organs. Mercurial frictions are, in the mean time, not to be neglected—"by combining these means, we shall often obtain the most unlooked for success." The following case is introduced as an example.

*Case.* F. P. aged 7 years, has complained for five days of sore throat, with swelling of the glands in the neck, fever, &c. On the sixth day the symptoms had rather abated. On the 7th day, the cough became very troublesome, and, towards the evening, it was croupal, the expectoration copious, glairy, and frothy, presenting fragments of croupy concretion, evidently from the larynx. There was now orthopnœa, sibilant inspiration, alteration of the voice. On examination, it was found that the throat was coated with a croupy concretion, of a whitish yellow colour. *Four grains of calomel were ordered every hour.* After the fourth dose, the expectoration was freer and more copious, and, in a fit of coughing, a membranous tube, three inches in length, was thrown up. The breathing, after this, was more free. Two alvine evacuations. *2nd day of treatment.* The breathing is again very difficult—somnolency—livid tint of countenance—incipient asphyxia. Mercurial frictions, with the strongest ointment, were employed every three hours, on the arms, neck, and chest. There was now great nervous agitation, convulsive cough, and another expulsion of a large quantity of concretion. The anterior portion of the tongue is clearing fast. *3d day of treatment.* The cough is less hard, the breathing easier. *Two grains of calomel every hour.* *4th day.* Ten frictions and 120 grains of calomel had now been used in about 50 hours. The gums were slightly affected—the breathing is still freer—the countenance nearly natural—the expectoration more bland—the voice clearer. In three days more the convalescence was complete, and no relapse followed.

"It is with a facility like this that, most commonly, the calomel modifies the diphtheritic inflammation, even when it has invaded the larynx and trachea. Such, also, is the rapid march of convalescence. But if the patient be exposed to cold, if the mercury be pushed too far, then we have a formidable train of symptoms ensue, and severe ulcerations of the skin and mucous membrane may follow." In the severe cold of February, 1826, Dr. Bretonneau saw these effects produced in three patients who were deprived of the comforts of life, and of sufficient protection against the rigour of the season. When the patient's circumstances are more favourable, the physician may, with common precaution, avoid all the bad consequences of the mercurial influence, and secure to his patient all its beneficial effects.

*Tracheotomy.* We now come to an interesting question. Are we, when all medicinal agents fail in giving relief, to sit down quietly and see the patient expire from a mechanical impediment to the function of respiration? Some efforts which have been made in the cause of humanity forbid this supineness. M. Bretonneau has performed tracheotomy, under such circumstances, in three instances, and in one with success. The Count de Puysegur had already lost three of his children by this dangerous malady, and the fourth was seized with the disease.

*Case.* Eliza de Puysegur, aged four years, of delicate constitution, but who had enjoyed good health, with the exception of a tertian fever which she had a year previously, was seized, on the 15th of June, 1825, in the morning, with dry cough, which increased towards mid-day, accompanied by swelling of the tonsils. 16th. The same symptoms; but in the night there was fever, with a barking cough. 17th. The breathing was noisy during sleep—the cough not so frequent. 18th. The little patient was brought from the country into town, a distance of 30 miles, and got some cold on the journey. Dr. Bretonneau was summoned to see her. She had now no fever, her appetite had not failed, and the cough was not very troublesome, but increased in the course of the night. 19th. A white patch on the left tonsil. *An emetic mixture.* In the evening the tonsils were swelled, and rather red. On the left there was an oblong patch bordered with redness, on the centre of its surface—lymphatic glands at the angle of the jaw swelled—cough short and dry. The hydrochloric acid was applied, by means of a sponge, to the tonsils, which assumed the usual white colour on being touched. The action of vomiting then took place. 20th. At three o'clock in the morning, the cough had become very much increased, but was accompanied by some expectoration of mucus. Two grains of calomel were given every two hours. At midday, there being some colicky pains, castor oil was given, and produced evacuations both upwards and downwards. In the evening the cough was looser, the deglutition easy, and there was no fever. The night was calm. 21st. Several stools to-day—cough loose. Some confectionary was given to the little patient this day. In the night the cough became dry, short, and frequent. Yet the fauces were pale, and the spot on the tonsil not extended. The voice is altered. Three grains of calomel, in three doses, given in the night. As the epidemic was not raging at this time, Dr. Bretonneau did not consider the disease as true diphtheritis, and was thus thrown off his guard. He did not prescribe calomel in doses proportioned to the danger which was at hand. 22d. (7th day of the disease.) At four o'clock this morning the inspirations became croupal, the lips of a violet colour, the cough convulsive, and followed by the exspuition of a membraniform concretion, ten lines in length, bifurcated, tenacious, and elastic. The pharynx had the same appearance as yesterday. The cough was decidedly croupal. Two grains of calomel every hour. The cough became more and more distressing, the inspirations convulsive, and the muscles on the front of the neck in violent action. The calomel was now given every half hour, and twenty-two grains were thus administered. Another and a much larger portion of concretion was thrown off by the cough, its form and dimensions leaving no doubt that it came from the trachea. The difficulty of respiration was not diminished by this expulsion. The calomel was given at still shorter intervals, and mercurial frictions were added. The inspirations became hourly more stridulous, and the action of the muscles on the neck more strenuous. Two grains of the polygala. A blister was applied to the larynx, and, as Dr. Bretonneau anticipated the operation of tracheotomy, care was taken not to allow the vesication to go below the thyroid region. The danger now became more and more imminent. Somnolency was fast increasing, in spite of the cough. The neck was swelled—the face livid, with every symptom of incipient asphyxia. The operation could no longer be deferred, and judging from the two former trials of this desperate remedy, sanguine hopes could not be held out to the agonized parents. In the two cases here alluded to, Dr. Bretonneau had made use of a small gum-elastic tube, which did not at all succeed in admitting and giving vent to a sufficient quantity of air. To this he attributed, and probably with reason, the fatal issue of the cases. In the present instance, he provided himself with a proper silver tube, of larger calibre, and then proceeded to the operation. The steps of this we need not detail, as they are familiar to surgeons in this country, in consequence of the comparative frequency of its performance here. The operator was exceedingly embarrassed by the convulsive action of the muscles, and the hæmorrhage from the thyroideal veins, some of which were as large as a crow-quill. The hæmorrhage at length being restrained, five rings of the trachea were divided, and the silver canula introduced. The breathing was instantly relieved. The canula was secured, and the quantity of blood lost was estimated at six ounces. Immediately after the operation, the child made signs for drink, and carried the cup to its own mouth, without assistance. A quantity of bloody mucus was ejected through the tube every time the cough was excited, and our author saw with satisfaction some long strings of the pellicular concretion mingled with the mucus. The night was spent quietly. The cough was not so frequent, and was always accompanied by expulsion of membraniform shreds. The child had two hours sleep. *Eight grains of calomel were blown in through the canula into the trachea. 2nd day after the operation.* The breathing is again become noisy, short, difficult, and accompanied by great muscular exertion—pulse accelerated. It was perceived that the calibre of the tube was diminished by mucosities, and the calomel adhering to its internal surface. It was withdrawn, cleaned, and replaced without difficulty. The breathing immediately became easier, and the pulse reduced in frequency. *3d day.* The patient has had tranquil sleep till 2 o'clock this morning, when the cough became troublesome, and some fragments of croupy concretion were expelled through the tube. The canula was again withdrawn and cleaned as before. Its introduction was this time very difficult. *4th day.* The cough was troublesome in the night—the tube became displaced, and was re-introduced with the greatest difficulty. Much membranous concretion thrown out during these trials. One piece of membrane which came away bore the impression of the interior of the glottis. The breathing became easier after this. *5th day.* The night was spent pretty quietly. The tube was displaced once or twice, and, while out, it was ascertained that some air was inspired and expired by the natural passage. Some violent convulsive coughing came on to-day—the tube was withdrawn—and several fragments of concretion came away. *6th day.* The air passed this day freely through the larynx when the tube was stopped or withdrawn. The canula was kept out for half an hour. We need not detail the case any farther. The tube was dispensed with on the 10th day after the operation, but large quantities of croupy membrane continued to be discharged by coughing for many days in succession. The little patient recovered.

Dr. Bretonneau has only been able to find one well authenticated instance on record where tracheotomy was successfully performed in croup. This was a case by Mr. Andree, which happened about the year 1782. We do not now remember where this case is recorded. In the 6th volume of the Medico-

Chirurgical Transactions, the late Mr. Chevalier published his case of bronchotomy in a child seven years of age. The little patient had thrown off a quantity of the membranous concretion; but, on the following day, he was on the point of suffocation, and Mr. C. performed the operation, which gave immediate relief, and the child recovered. In the second volume of our *analytical series,* (Dec. 1821) we made our readers acquainted with Mr. Carmichael's two cases of tracheotomy, one of which was successful. They were, however, in adults and for laryngitis. In the same volume of our Journal, page 878, we introduced a successful case of tracheotomy by Mr. Porter of Dublin, for the relief of laryngitis. In the second volume of our present series, p. 215, we gave a case of croup in an adult, successfully operated on by Mr. Hume, of Coventry—and in the same volume, p. 223, a successful operation of tracheotomy, by Mr. Carmichael, of Dublin, in a case of laryngitis. Mr. Goodeve, of Clifton, lately performed tracheotomy with success, in a case of ulcerated larynx. The tube was worn for six months afterwards. Drs. Denmark and Johnson have related a remarkable case, where the tube still continues to be worn by the patient, after a lapse of ten or eleven years. There are some other cases of this kind on record; but these are the principal ones that at present occur to our memory. They are sufficient, in connexion with the very important case which we have translated from Dr. Bretonneau, to induce a hope that tracheotomy will be more frequently resorted to in croup than formerly.

We cannot pass over in silence the curious coincidence pathological and therapeutical, between Dr. Bretonneau and Mr. Mackenzie, of Glasgow. Mr. Mackenzie not only avers that the croupy exudation very frequently begins on the tonsils, and spreads thence to the larynx and trachea; but he proposes the very same remedy—caustic to the incipient inflammation. The Frenchman uses muriatic acid—the Englishman argentum nitratum. As far as *ex professo* publication goes, Mr. Mackenzie has the claim to priority—his observations appearing in April, 1825. But it must not be concealed that Dr. Bretonneau's memoir was read at the Royal Academy of Medicine in the year 1821—and what is still more important, that M. Guersent had, long previously to Mr. Mackenzie's publication, given an outline of Dr. Bretonneau's pathology and practice in the New Dictionary of Medicine, under the article *Angine Couenneuse,* and also *Croup.* These facts, we fear, must not only deprive our countryman of the claim to priority, but induce some suspicion that Mr. Mackenzie, who is intimately acquainted with all continental writings, could hardly be ignorant of the doctrine and therapeutics of Dr. Bretonneau, at the time he published his paper in the Edinburgh Journal. Mr. Mackenzie hints, indeed, that he received his information respecting this peculiar treatment of croup from some other person—but who this person is, he does not inform us. The present article will probably induce him to be more explicit on this point.

The work which we have just analyzed we consider as interesting; and we hope it will lead practitioners to examine with attention the primary phenomena of croup, in order to ascertain whether or not it so frequently commences on the surface of the tonsils as Dr. Bretonneau and Mr. Mackenzie affirm. It will also lead them to pay great attention to what is called malignant or ulcerated sore throat, since it is fairly shown that the inflammation often spreads to the larynx, and there proves a dangerous or fatal disease. If the topical applications recommended by these two authors prove as useful in the hands of others as in their own, the present article will not be destitute of interest, or unattended by benefit to the profession and to society at large. Under this impression, and with this anticipation, we commit it to their examination.

